# Genetic Association Studies: An Information Content Perspective

**DOI:** 10.2174/138920212803251382

**Published:** 2012-11

**Authors:** Cen Wu, Shaoyu Li, Yuehua Cui

**Affiliations:** 1 Department of Statistics and Probability, Michigan State University, East Lansing, Michigan 48824; 2 Department of Biostatistics, St. Jude Children's Research Hospital, Memphis, TN 38105; 3 Center for Computational Biology, Beijing Forestry University, Beijing, China 100083

**Keywords:** Conditional entropy, Entropy, Gene-centric analysis, Haplotype analysis, Mutual information, Epistasis, Synergistic effect, Single nucleotide polymorphism.

## Abstract

The availability of high-density single nucleotide polymorphisms (SNPs) data has made the human genetic association studies possible to identify common and rare variants underlying complex diseases in a genome-wide scale. A handful of novel genetic variants have been identified, which gives much hope and prospects for the future of genetic association studies. In this process, statistical and computational methods play key roles, among which information-based association tests have gained large popularity. This paper is intended to give a comprehensive review of the current literature in genetic association analysis casted in the framework of information theory. We focus our review on the following topics: (1) information theoretic approaches in genetic linkage and association studies; (2) entropy-based strategies for optimal SNP subset selection; and (3) the usage of theoretic information criteria in gene clustering and gene regulatory network construction.

## 1 INTRODUCTION

The availability of high-density single nucleotide polymorphisms (SNPs) generated routinely in many labs provides unprecedented opportunities in understanding the genetic machinery of many complex human diseases. Following the completion of human HapMap project and the development of high-throughput genotyping biotechnologies, SNPs have become the primary source to unravel the genetic roots of complex diseases which has led to the flourishing of vast amount of SNP data analysis methodologies [1]. Genetic association analysis based on SNPs has been traditionally focused on single SNP analysis. Methods of single SNP analysis include but are not limited to simple chi-square test, logistic or linear regression analysis depending on the underlying disease trait distribution [2]. These analyses consider one SNP at a time, followed by multiple testing corrections in a genome-wide scale. Single SNP-based analysis has been broadly applied in early stage genome-wide association studies (GWAS). For a complex disease, it often involves multiple genetic variants functioning jointly, each with a small or moderate effect. Thus, it is likely that the single SNP-based analysis may miss many potential players due to their small marginal effect size to reach a genome-wide significance level. Given the complexity of many human diseases, single SNP-based analysis certainly has its limitations. An alternative way to look for association is to analyzing a group of SNPs simultaneously through haplotype analysis. The advantage of haplotype-based analysis over single SNP-based approach has been investigated [3-5]. The biological interpretation is that multiple functional alleles in a *cis* position within a gene can function jointly to make a “super allele” with a large effect on disease phenotypes [6]. However, the phase ambiguity problem presents computational challenges for haplotype-based analyses. Algorithms have been developed to infer haplotype frequencies such as fastPHASE [7]. The estimated frequencies can be treated as covariates in a linear or logistic regression framework to infer specific haplotype effects [6, 8, 9]. 

Despite great successes achieved by GWAS so far, only a small proportion of the heritability of complex traits has been explained [10, 11]. One possible explanation is that methods based on univariate analysis can only identify variants with strong marginal effects, leaving for large proportion of heritability unaccounted for due to potential interactions among genetic variants [12]. This leads to a broad discussion of analysis at different levels such as the gene-based analysis [13-15], gene-set analysis [16] (reviewed by Fridley and Biernacka 2011), and pathway-level analysis [17] (reviewed by Wang 2010). These analyses at different levels are essentially to capture the overall evidence of association of a set with a phenotype, which may contain potential interaction between multiple variants in the set. However, these analyses are essentially different from traditional gene-gene interaction (GGI) analysis in a statistical sense. In a typical GGI analysis, interacting variants are analyzed through either parametric models such as linear or logistic regression, or nonparametric methods such as multifactor dimension reduction (MDR) [18]. Until recently, Li and Cui proposed a gene-centric gene-gene interaction analysis which combines gene-based analysis and interaction analysis into a unified framework and provides additional insight into disease etiology that cannot be discerned from single SNP-based interaction analysis [19].

With the development of next-generation sequencing technology, more dense SNP markers are generated, raising the hope for the discovery of rare variants associated with complex diseases. However, current bottleneck in genetic association studies is not limited by genotyping technology, but rather limited by high computational cost and the lack of efficient and powerful statistical methods. The underlying gene action mode is never clear to us, leaving us great difficulty in choosing right models to fit the data. The information theory, which initially emerged in the 1940s [20] to quantify the transmission of information in communication channels within a rigorous mathematical framework, has gained much attention in genetic association studies recently [e.g.,  15, 21-23]. In this paper, we give a brief review of information theory-based genetic association studies including the applications in single SNP-based, haplotype-based, gene-based, gene-gene interaction, gene-environment interaction, gene clustering and gene network construction analysis. We describe key challenges in these areas, how information-based approaches fit in the framework as potential solutions, the unsolved issues and directions for future work. 

## 2 BACKGROUND ON INFORMATION THEORY

We present some key concepts of information theory in this section. Information theory quantifies the uncertainty of a random variable by its entropy section [24]. Given a discrete random variable (r.v.) *X* with a set of possible values {*x_1_*, *x_2_*, *x_n_*}, and corresponding probability distribution *p*(*x_i_*) = *P* (*X* = *x_i_*), *i* = 1, 2, ..., *n*, entropy of *X* is defined as

(1) $$H(X)=-\sum \mathrm{ip}\left(x_{i}\right)\log_{b}p\left(x_{i}\right)$$

The higher the entropy, the higher the uncertainty we may predict the outcome of *X* and the entropy is maximized when all the possible values of *X* are equiprobable. When using the base 2 logarithm (*b* = 2), the entropy is measured in bits. 

Joint entropy of two discrete random variables *X* and *Y* is defined analogously as,

(2) $$H\left(X,Y\right)=-\sum_{i}\sum_{j}p\left(x_{i}y_{j}\right)\log_{b}p\left(x_{i}y_{j}\right)$$

where *p*(*x_i_*, *y_j_*) = *P*(*X* = *x_i_*, *Y* = *y_i_*) refers to the joint distribution of the two variables. An important property of entropy is subadditivity: *H*(*X*, *Y*) ≤ *H*(*X*) + *H* (*Y*), equality holds if and only if *X* and *Y* are statistically independent. 

Mutual information is defined as the information common to both random variables,

(3) $$I\left(X;Y\right)=H\left(X\right)+H\left(Y\right)-H\left(X,Y\right)\geq 0$$

*I* (*X*; *Y*) provides the information of *X* contained in *Y*, or vice versa. *I* (*X*; *Y*)= 0 when *X* and *Y* are statistically independent. The uncertainty remaining in r.v. *X* given the knowledge of r.v. *Y* is defined as the conditional entropy

(4) $$H\left(X|Y\right)=H\left(X\right)-I\left(X;Y\right)=H\left(X,Y\right)-H\left(Y\right)$$

Similarly, the uncertainty remaining in r.v. *Y* given the knowledge of r.v. *X* is measured by conditional entropy

(5) $$H\left(X|Y\right)=H\left(Y\right)-I\left(X;Y\right)=H\left(X,Y\right)-H\left(X\right)$$

From (3) we have, *I*(*X*;*X*) = *H*(*X*), i.e. the mutual information of a r.v. *X* with itself indicates the decrement in the uncertainty of r.v. *X* given the information of itself. Thus entropy is also referred to as self-information. Fig.(**1**) shows a Venn diagram of *I* (*H*; *Y*), *H* (*X*), *H* (*Y*). The left and right ellipses indicate the magnitude of the marginal entropies *H* (*X*), *H*(*Y*), respectively, and their overlap corresponds to the magnitude of the mutual information *I* (*X*; *Y*).

As an illustrative example of entropy in genetic studies, let’s consider two bi-allelic SNP markers. Suppose alleles of the two SNPs (SNP A and SNP B) are A, a and B, b, respectively, so there are three genotypes at the two markers. Denote genotypes at marker A as *X* and genotypes at marker B *Y* as, then

$$X=\left(\begin{array}{ccc} \mathrm{AA} & \left(2\right), & p\left(2\right)\\ \mathrm{Aa} & \left(1\right), & p\left(1\right)\\ \mathrm{aa} & \left(0\right), & p\left(0\right) \end{array}Y=\left\{\begin{array}{ccc} \mathrm{BB} & \left(2\right), & q\left(2\right)\\ \mathrm{Bb} & \left(1\right), & q\left(1\right)\\ \mathrm{bb} & \left(0\right), & q\left(0\right) \end{array}\right.\right.$$

*p*(·), *q*(·) denote corresponding genotype frequencies. The marginal entropy of marker A and marker B are then defined by

$$\begin{array}{c} H\left(X\right)=-\sum_{i=0}^{2}p\left(i\right)\log_{2}p\left(i\right)\\ H\left(Y\right)=-\sum_{i=0}^{2}q\left(i\right)\log_{2}q\left(i\right) \end{array}$$

And the joint entropy of the two markers is

$$H\left(X,Y\right)=-\sum_{i=0}^{2}\sum_{j=0}^{2}P\left(X=i,Y=j\right)\log_{2}P\left(X=i,Y=j\right)$$

Then the mutual information and conditional entropies can be calculated accordingly using (3), (4) and (5). 

## 3 ENTROPY-BASED LINKAGE AND ASSOCIATION STUDIES

### 3.1 Single SNP-Based Association Studies

One of the major issues of single-SNP based analysis is little power in detecting variants with small/moderate effect size or SNPs with small minor allele frequency (MAF). Entropy takes the nonlinear transformation of allele frequencies. When testing for allele frequency difference between cases and controls, this nonlinear transformation can potentially increase the testing power [25, 26]. With this property, the entropy-oriented approaches have drawn public attention in association tests. Kang and Zuo [21] explored the performance of the entropy-based statistic in the two-stage genome-wide association studies. When minor allele frequency (MAF) is small, a direct test of MAF difference often ends up with lower power. Their investigation shows that the power of the entropy-based joint analysis is larger than the linear joint analysis under a large range of MAF differences between cases and controls. Ruiz-Marín [27] proposed a new allelic entropy test from the symbolic dynamics point of view. They showed the consistency of the test for a broad spectrum of alternative hypothesis and demonstrated the efficiency of the test toward SNPs with MAF between 1% and 5%. This investigation shows the advantage of the entropy-based test in detecting SNP variants with small MAF.

### 3.2 Haplotype-Based Association Studies

Haplotypes are combinations of marker alleles that are closely linked on the same chromosome and are inclined to inherit as a unit. They are essential to decipher the genome-wide human LD pattern. In the association study of complex disease gene mapping, the haplotype-based approaches are statistically more appealing than those single locus based approaches in certain scenarios. Haplotype association analysis can be conducted under a population-based or family-based design [28]. Considering the benefits of haplotype-based analysis, Zhao [23] proposed an entropy-based approach which is shown to improve the power of the standard χ^2^ test for GWAS via the nonlinear transformation of haplotype frequencies. Furthermore, Zhao [26] systematically investigated four types of nonlinear tests, the test statistics of which are in the form of entropy, exponential function, quadratic function and reciprocal function. They also showed that the similarity measure-based statistics in Tzeng [29] are equivalent to the nonlinear test statistics. This study demonstrates the power of entropy-based test in haplotype analysis. In summary, entropy-based approaches tend to outperform their non-information theoretic counterparts even through high computational cost in inferring haplotype phase and frequencies could hinder the development of haplotype-based analysis.

### 3.3 Gene-Based Association Studies

Genes are the functional units in most living organisms. The sequence information and function of genes are highly consistent across diverse populations, which makes the gene-based association study more robust in terms of replication compared to the single SNP-based analysis [13]. Moreover, genic SNP variants are more likely to alter gene function and affect disease risk than those that occur outside genes [14]. Thus, association analysis by focusing genes as testing units is biologically attractive by reducing the genotyping cost. In addition, it is statistically appealing by reducing the number of tests in a genome-wide scale [13]. Applying the entropy measure as a tool for nonlinear transformation of joint genotype frequencies, Cui [15] developed a gene-centric genome-wide association test. This method defines entropy on joint genotype frequencies in cases and controls and is computationally faster than the haplotype-based entropy test proposed by Zhao [23]. Simulation studies have shown the superior power of the entropy-based analysis under a number of scenarios. The underlying principle for the two methods is essentially the same, that is, to capture the multilocus linkage disequilibrium (LD) information in a region. Cui [15] defined a penalized entropy measure in order to group rare joint genotypes into common ones and therefore save computational time. Generally speaking, entropy test based on joint genotypes is computationally more attractive, especially when large number of SNPs are involved in a gene region. 

Cui ’s method can be easily extended into other scenarios. For example, one can consider multiple SNP variants that are physically located close to each other on a chromosome band, or one can extend the gene idea to a set of genes or pathways and assess the association from a gene-set or pathway level. The gene-set or pathway information can be pre-defined, such as from the KEGG pathway [30] or Gene Ontology database. By incorporating various levels of information into an association test, we could gain additional biological insights into the disease etiology of a complex disease. These extensions can be done by the entropy-based test with properly selected grouping threshold to control false positives [15]. 

### 3.4 Family-Based Association Studies

In population-based association studies, all samples cannot be guaranteed to come from a homogeneous population. When individuals are sampled from different populations and if different populations have different disease allele frequencies, false positive or false negative could be observed if the population substructure is not properly taken care of in the analysis [31]. This problem can be avoided by adopting a family-based design through which parents are treated as controls [32]. One of the commonly used methods in a family-based association study is the Transmission Disequilibrium Test (TDT) [33]. TDT measures association in families with transmissions of risk markers from parents to offspring in a nuclear family. Under the condition that an allele is associated with a disease trait, it will be transmitted to the affected offspring more often than expected by chance and this can be detected by the TDT test. Zhao [34] generalized the original TDT statistics, which are linear functions of transmitted allele frequencies, into the nonlinear ones via entropy. The authors examined the distribution of the novel TDT test statistics in both homogeneous and admixture populations and demonstrated the entropy-based test is more powerful than the original TDT test. The entropy-based TDT test offers an alternative powerful solution to improve the traditional TDT test in the population-based association studies. 

### 3.5 Entropy-Based QTL Mapping

Quantitative trait loci (QTL) mapping has been a powerful tool in elucidating the genetic basis of complex traits [35]. In a typical QTL mapping study, all samples are utilized following a particular genetic design in plants and animals, or in a family- or population-based human genetic study. Due to various issues for sample collection, selective genotyping provides a cost-saving solution. Under this strategy, one tests the differences of allele frequencies between an upper tail and a lower tail in a mapping population. Thus, the non-linear transformation of allele frequencies via Shannon entropy can be applied to boost the mapping signal. Li [36] extended the entropy-based test to an association analysis by embedding the test statistic of Zhao [23] in the scenario of extreme individuals of a population. A potential application of this strategy is in eQTL mapping in which selective transcript profiling is often conducted due to high cost. 

### 3.6 Entropy-Based Gene-Gene and Gene-Environment Interaction Studies

It is commonly recognized that gene-gene (G×G) interaction or epistasis, and gene-environment (G×E) interaction play key roles in determining the risk of complex human diseases [37, 38]. The presence of epistasis among susceptible disease loci may lead to the detection of causal variants or else undiscovered [37], while taking G×E interaction into account can gain additional insight into the etiology of complex diseases. For a comprehensive review of G×G interaction in the context of human genetic association studies, readers are referred to Cordell [39].

From an information content perspective, Moore [40] developed a general multistep strategy to investigate the epistasis in complex disease studies. They evaluated the information gain through entropy measure in order to choose a subset of appealing SNPs, and then simplify the subset into single attribute that seizes the nonlinear interaction information among multiple SNPs. Dong [41] defined disease entropy in the form of,

Hp,1−p=−p log p−1−p log1−p

where *p* and (1 - *p*) are the proportions of cases and controls in a sample, respectively. Then the disease entropy condition on a SNP can be calculated by adding the sub-entropy corresponding to all the possible genotypes. This approach not only can discover the G×G interaction effects by a package called ESNP2-S as most of the other method did on epistasis, but also can identify the optimal model from all two-locus genetic interaction models. For a case-only design, Kang [42] developed an entropy-based test statistic for interaction analysis on multiple loci. The new test is biologically more attractive in the sense of detecting causal loci associated with a clinical phenotype. 

For the study of G×E interaction, Wu [43] investigated the performance of the mutual information-based test statistic in G×E interaction studies, assuming independence between a gene and environment variable. They developed the global test statistic *T_GE_* to test gene-environment interaction, and *T_G_i_E_* to test the genotype and environment interaction. They showed that the information theoretic approach is more powerful than the logistic regression approach. They found that p-values calculated from *T_GE_* are smaller than those obtained by *T_G_i_E_* in general, which is due to the difference in the amount of information used by *T_GE_* and *T_G_i_E_*. This evaluation provides a new framework for the study of G×E interaction and could be extended to a genome-wide association analysis. Fan [25] proposed entropy-based information gain test statistics to detect and characterize G×G and G×E interactions of complex diseases. The authors proposed one-dimensional test statistics for testing three-way and higher order interactions. Their study indicates that the information gain based test statistics have similar or higher power compared to naïve chi-square test and are more robust. 

### 3.7 Detecting Synergistic Effect with Information Theory

To identify a set of variants in a gene, a gene set or a pathway associated with complex diseases, one needs to model the joint effect of those variants as a system. Synergy analysis provides a novel strategy for this purpose. The bivariate synergy between two SNPs *X*_1_ and *X*_2_ with regard to the phenotype *Z* is defined as,

(6) $$\mathrm{Syn}\left(X_{1},X_{2};Z\right)=I\left(X_{1},X_{2};Z\right)-\left[I\left(X_{1};Z\right)+I\left(X_{2};Z\right)\right]$$

It quantifies the additional information conveyed by the cooperative effects of the two SNPs with respect to the information conveyed by the two separately. Positive synergy indicates the existence of the synergistic effect or the interactions of all the SNPs in a genetic system, while negative synergy indicates information redundancy, hence joint analysis may not be preferred. Schneidman [44] defined zero synergy as information independence. Synergy also quantifies the mutual information gain of two SNPs to a phenotype, thus Eq. (6) can be rewritten as,

(7) $$\mathrm{Syn}\left(X_{1},X_{2};Z\right)=I\left(X_{1};X_{2}|Z\right)-I\left(X_{1};X_{2}\right)$$

If the quantities *I*(*X*_1_; *X*_2_ | *Z*) and *I*(*X*_1_; *X*_2_) are zero, then the relations between *X*_1_ and *X*_2_ are defined as conditional independence and activity independence, respectively [44]. Note that the information independence can hold without the conditional and activity independence when *I*(*X*_1_; *X*_2_ | *Z*) and *I*(*X*_1_; *X*_2_) are equal. Schneidman [44] systematically examined the interrelated framework of the three measures of independence and correlation. Varadan [45] later on extended the synergy definition to a set of multiple SNPs {*X*_1_; *X*_2_ ... *X*_n_} and a phenotype *Z* as,

SynX1,X2..., Xn;Z=IX1,X2..., Xn−max all partitions intoSi∑iISi,Z

where the partition is denoted as U*_i_* *S_i_* = {*X*_1_, *X*_2_ ... *X*_n_} and ∩*_i_* *S_i_* = ø. The generalization from bivariate synergy to multivariate synergy is consistent with the intuitive idea that synergy is the extra information conveyed to the phenotype by comparing the information contribution between the entire set and the maximum information partition of the entire set. 

Anastassiou [46] elaborated the synergy tree and showed it is connected to the pathway analysis. The positive synergy in this scenario implies the interaction among pathways. The main advantage of the synergistic analysis lies in pinpointing the pathway structures related to the genotype. The sets of interacting genes can be identified without the biological knowledge which is needed afterwards to verify the consistence between the identified genes and known pathways. The author also pointed out two restrictions of analysis of synergy, the incapability to locate the causal high synergy sets to the phenotype, and the limitation to identify the overlapping pathway structures because of the disjoint sets after partition. 

The synergy analysis opens an alternative framework for the evaluation of G×G interaction. Under the gene-based association analysis (e.g., [15]), all variants within a gene or a gene set are included to define a gene signal. This, however, has great limitations when noisy variants are not properly taken care of in the defined entropy statistics. They could inflate the degree of freedom for a test statistic and consequently affect testing power. An optimal strategy is to do a two-stage selection in which one can select potential SNPs by evaluating their synergy. Also one can assess the joint synergy to determine whether to implement a gene- or set-based association test. If the joint synergy is less than zero, it implies that the joint analysis does not give much additional information, hence a single SNP-based analysis may be preferred. 

## 4 TAG SNP SELECTION USING ENTROPY

SNPs are the most abundant genetic variants in human genome. The emergence of the high-throughput genotyping technology has made the fast genome-wide SNP association analysis possible. The genotyping cost is no longer prohibitively high with the advancement of the high throughput genotyping techniques. Nevertheless, serious multiple testing problems are triggered when all recognized SNPs are genotyped. Recent studies have revealed that SNPs in the human genome are distributed as block like patterns [47, 48]. SNPs within each block are strongly correlated due to high linkage disequilibrium (LD). Thus, haplotype diversity within a block can be well explained by a small number of SNPs, called tag SNPs (tSNPs) [48-50]. The existence of these tSNPs implies the un-necessity to associate a disease with all SNPs in the genome to understand its genetic etiology. On the other hand, focusing only on these representative tSNPs can greatly facilitate genetic association study with reduced cost and false negatives (by alleviating the multiple testing burden). Thus, the selection of tSNPs becomes critically important both in cost and statistical inference for association analysis.

Halldórsson [51] commented that methods for selecting tSNPs should consist of three major steps: (1) determining the structure of the predictive genomic regions; (2) Evaluate the quality of the tSNP subsets; and (3) Reduce the number of tSNPs to the lowest possible level. A number of algorithms have been developed to identify tSNPs (e.g., [52-54]). However, many existing algorithms step-wisely add SNPs to a subset and have not well taken the 3^rd^ step into consideration. For example, those summarized in (Table **1**) of Weale [55] select tSNPs according to certain criteria without providing the guidance on how to sensibly choose minimal number of tSNPs.

Commonly used criteria for evaluating tSNP subsets within a haplotype block includes: the ratio of the haplotype diversity explained by the subset, and the association between the complete set and the tagging set, which is measured by *r^2^*, the coefficient of determination [55]. The second criterion examines the performance of the subset in terms of predicting the untagged SNPs, hence statistically more appealing. Nothnagel [56] first time introduced information theory to extend the classical pairwise LD measure to multi-locus version. They proposed the normalized entropy difference, ε, to measure the LD strength among multiple loci and locate haplotype blocks. A similar measure was proposed by Zhao [57] based on the likelihood theory. Liu and Lin [58] claimed that the performance of ε under two loci case cannot be compared with the traditional pairwise LD measures due to the incapacity of ε to attain the upper bound, and this ε metric may not detect haplotype blocks with limited number of SNPs. Defining *X* as a random variable of haplotype and *X_j_* as a random variable of allele at the *j*th SNP, Liu and Lin [58] proposed an entropy-based criterion to weight a normalized haplotype diversity measure by

$$\mathrm{HD}\left(S\right)=\frac{H\left(S\right)}{H\left(X\right)}$$

and a normalized LD measure by

$$\mathrm{ER}=\frac{E}{\sum_{j=1}^{n}H\left(X_{j}\right)-\mathrm{maxH}\left(X_{j}\right)},$$

where *H*(*S*) and *H*(*X*) are the joint entropy of the subset and the complete set, respectively; and *E* is the relative entropy. It has been shown that *ER* successfully captures the pattern of complete LD. In contrast to the above two measures which estimate the LD pattern across a whole chromosome region, Zhang [59] developed the metric MIR to infer the pattern between two arbitrary genomic regions and asserted that this metric outperforms pairwise and other multilocus metrics in terms of detecting haplotype blocks and selecting tSNPs. 

In addition to the above mentioned tSNP selection methods using information theory, several other works have also shown the benefit of using entropy in tSNP selection. Hampe [60] fused the SNP diversity, distribution and the corresponding haplotype structure into a single utility metric to choose informative SNPs. They showed the usefulness of the entropy-based metric in selecting tSNPs, even when the location and frequency of the disease allele is not available. Su [61] derived cost functions with information measures based on the genome-wide haplotype block structure. The authors devised an iterative partition-inference (IPI) algorithm to parse haplotype blocks and infer missing SNPs simultaneously, whereas the threshold of partitioning blocks is determined by an entropy map. 

Although informative SNP selection based on haplotype block structure has been intensively studied, Halldórsson [62] argued that the agreement on defining haplotype block boundaries and assessing the block quality has not been reached. The latent LD in a sample cannot be fully captured by the block structure and the block-to-block LD is disregarded. To overcome those drawbacks, Halldórsson [62] developed a block-free method, called K-MIS, which tremendously reduces the number of tSNPs compared to the block dependent methods. Liu [63] later on designed an entropy based iterative Cross Entropy Monte Carlo (CEMC) algorithm to select tSNPs, which has been shown to be superior over K-MIS algorithm. Even though the information-based tSNP selection methods have not been the major player in real application, they do show relative merit in certain special cases and deserve more investigation. 

## 5 APPLICATIONS OF THEORETIC INFORMATION CRITERIA ON GENE CLUSTERING AND NETWORK INFERENCE

Information theory has also been applied in a number of other applications in genetics and genomics study. For gene clustering analysis, Zhou [64] proposed a mutual information minimization algorithm by minimizing the sum of pairwise mutual information (PMI) between arbitrary two groups for the purpose to construct gene regulatory networks from expression data. The authors later on improved their clustering algorithm by combining metric of mutual information and fuzzy membership and achieved best performance [65]. The methods utilize the property that mutual information can measure dependence among gene random variables. 

For network inference, Margolin [66] designed an algorithm for the reconstruction of accurate cellular networks (ARACNE), which first time used the data processing inequality (DPI) from the information theory to significantly reduce potential false positive interactions in network deconvolution. Qiu [67] later on dramatically improved the efficiency of the pairwise mutual information calculation in ARACNE. Considering the limitation of mutual information in gene-gene interaction analysis, Zhao [68] proposed a conditional mutual information (CMI) measure to infer interactions among genes and further constructed gene regulatory networks. The algorithms they proposed can reduce the false positive connectivity rate. In particular, one of the algorithms is able to produce connectivity confidence by direct connectivity metric (DCM), instead of examining the dichotomous states of presence or absence of connections. Liang and Wang [69] proposed a conditional mutual information estimator using both discrete and continuous variables targeting networks containing both co-regulated and interactively regulated genes. As genes function in networks, construction of gene networks could shed novel insight into the function of genetic variants. Method development using theoretic information criteria in this area deserves more attention and investigation. 

## 6 CONCLUSION AND PROSPECT

Last decades has visualized the great success in genome-wide association studies with common SNP markers. Association analysis in an information content framework has achieved large success and caught researchers’ attention in the past. Its applications in a variety of areas in genetic and genomic studies have led to the advancement of our knowledge about the genetic machinery of complex diseases. With the next wave of genetic association studies in gene-sets or pathway levels, more novel variants are expected to be discovered and insightful interpretation of disease signals could be reached. However, how to deal with the every-increasing dimension of SNP variants and extend them to the gene- and/or pathway-based analysis represents daunting challenges. We have recently developed an efficient approach in selecting potential SNP variants and further incorporated them into a gene- or pathway-based analysis [70]. This selection process is different from the traditional tSNP selection discussed in section 4 since we incorporated the disease status information into the selection process. The selection procedure eliminates potential noisy SNPs having little or no contribution to a disease status, hence greatly improves the testing power for a gene- or pathway-based analysis.

The recent breakthrough in next-generation sequencing technology has made it possible to identify even more dense SNPs with a much larger range of MAF, especially small MAFs. These so called rare variants stimulate another wave of association analysis aimed to detect genome-wide rare variants contributing to common diseases [71]. With the demonstrated power and computational efficiency in analysis from an information theory perspective, more investigations and methodology development in related areas should be greatly encouraged.

## Figures and Tables

**Fig. (1):**
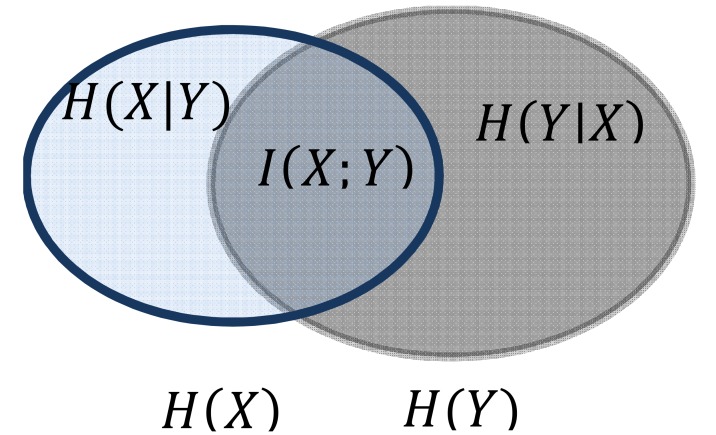
The Venn diagram of entropies *H*(*X*), *H*(*Y*), and the mutual information *I*(*X*;*Y*)
